# Resistance of storage roots of 46 sweetpotato cultivars to foot rot disease caused by *Diaporthe destruens* was evaluated using a laboratory test

**DOI:** 10.1270/jsbbs.25019

**Published:** 2025-08-20

**Authors:** Hiroaki Tabuchi, Akira Kobayashi, Yukari Kawata, Takeo Sakaigaichi, Keisuke Suematsu, Yuki Ohdaira Kobayashi

**Affiliations:** 1 Kyushu Okinawa Agricultural Research Center, NARO, Miyakonojo, Miyazaki 885-0091, Japan

**Keywords:** foot rot, *Diaporthe destruens*, sweetpotato, laboratory test, resistance, storage root

## Abstract

Despite the development of several cultural pest controls, foot rot disease has severely damaged sweetpotato crops in southern Japan since 2018. A more effective solution would be to breed resistant cultivars. To promote the breeding of cultivars whose stems are resistant to this disease, we previously developed a laboratory resistance test using sweetpotato stems. The laboratory test can indirectly evaluate the foot rot resistance of stems in the field test, which is usually used to evaluate cultivar resistance in breeding programs. In the present study, we improved laboratory test by using sweetpotato storage roots and investigated the resistance of storage roots of 46 cultivars. We analyzed the correlations of various indices of resistance of storage roots between the laboratory test and the field test, and found that the index of the laboratory storage root test correlated significantly with the proportion of severely rotted storage roots in the field test in some cases. This result indicated that the laboratory storage roots test could indirectly evaluate the foot rot resistance of storage roots in the field test. These two laboratory tests using stems and storage roots could help breed cultivars whose stems and storage roots are resistant to foot rot.

## Introduction

In 2018, sweetpotato stem withering and storage root rotting were frequently observed in the southern Japanese prefectures of Kagoshima, Miyazaki, and Okinawa, resulting in significant losses in commercial yields. Foot rot disease, caused by *Diaporthe destruens* (Harter) Hirooka, Minosh. & Rossman, was also observed in these prefectures (Kobayashi 2022). Because this was the first observation of foot rot in Japan, there was no information about effective cultural pest controls or resistant cultivars available in Japan. Various cultural pest control measures were tried. Nishioka *et al.* (2021) reported effective treatments to prevent the spread of foot rot from latently infected storage roots to uninfected ones during storage. Those treatments included harvesting storage roots from uninfested fields, selecting uninfected storage roots after washing soil off their surfaces to aid in detecting symptoms of foot rot, cutting root stalks off because they might be latently infected, and then disinfecting the roots with thiophanate-methyl before storage. Nishioka *et al.* (2021) also reported that steam treatment of seed storage roots at 48°C for 40 min extensively suppressed the spread of foot rot during storage without affecting germination potential. Saito *et al.* (2024) indicated that vapor heat at 48°C for 100 min disinfected storage roots inoculated with *D. destruens* by 93.8%. Nomiyama (2023) and Usui and Kushima (2020) indicated that anaerobic soil disinfestation of nurseries and benomyl treatment of stem cuttings, respectively, were effective in preventing *D. destruens* from being brought from nurseries to fields. Nomiyama (2023) infested a nursery with infected storage roots, treated it with anaerobic soil disinfestation, and placed healthy seed storage roots into the nursery. No stems with symptoms of foot rot were observed among stems germinated from the healthy seed storage roots for 5 months. Usui and Kushima (2020) prepared cuttings by cutting stems, which had been germinated from infected storage roots, at 5 cm above a symptom of foot rot. The cuttings were transplanted to pots. Symptoms of foot rot were observed in 40% of the cuttings without benomyl disinfection 127 days after transplantation. On the other hand, none of the cuttings disinfected with benomyl showed symptoms of foot rot 127 days after transplantation. Kobayashi (2022) and Shima *et al.* (2024) described effective treatments to prevent foot rot from spreading in fields: spraying a pesticide containing azoxystrobin 5 weeks after planting, removing plants with symptoms of foot rot promptly, spraying copper wettable powders just after removal of infected plants, draining water sufficiently after rainfall, and early harvesting of sweetpotato to remove host sources of foot rot from fields. After harvesting, effective treatments are the removal of plant debris, which are among the first infection sources in fields the following year, the disinfection of fields using dazomet or carbam sodium to prevent *D. destruens* from remaining in the soil, and rotation of sweetpotato and crops other than *Convolvulaceae*, since *Convolvulaceae* are the only known hosts of *D. destruens* (Clark and Moyer 2013, Kobayashi 2022, Shima *et al.* 2024, Usui and Kushima 2020).

Regarding the use of resistant cultivars, which is a low-cost management approach, field resistance tests were carried out in a field naturally infested with *D. destruens*, and some cultivars showed relatively high resistance (Kobayashi *et al.* 2025, Kyushu Okinawa Agricultural Research Center, NARO 2023). However, it was necessary to breed resistant cultivars for various uses because certain major cultivars (such as ‘Koganesengan’ and ‘Shiroyutaka’ for raw materials and ‘Beniharuka’, ‘Beniazuma’, and ‘Kokei No. 14’ for table use) do not have sufficient resistance to foot rot (Kobayashi *et al.* 2025, Kyushu Okinawa Agricultural Research Center, NARO 2023). To breed resistant cultivars, field resistance tests are carried out in routine breeding programs. However, problems have been found with these tests (Tabuchi *et al.* 2024): fungal density is not uniform, infested fields should be away from farmer’s fields, the test is done only once a year, and much labor is needed. We previously developed a laboratory resistance test using stem cuttings to solve these problems, enabling us to investigate the resistance of a stem throughout the year without being influenced by natural conditions (Tabuchi *et al.* 2024). In the present study, we report improved laboratory resistant testing using storage root slices and the results of the resistance of storage roots of 46 cultivars. We also analyzed and discussed the correlation of indices between laboratory tests and field tests.

## Materials and Methods

### Fungal and plant materials

We used the F3 strain of *D. destruens*, which was originally isolated from fields in Miyazaki prefecture in Japan (Nomiyama *et al.* 2022), as the foot rot pathogen in this study.

We investigated the resistance of storage roots of 46 sweetpotato cultivars, including the major cultivars in Japan, using the laboratory test (Tables 1, 2) (Ministry of Agriculture, Forestry and Fisheries 2024). They were cultivated in the fields at Kyushu Okinawa Agricultural Research Center, NARO, Miyakonojo, Miyazaki prefecture, Japan. Storage roots were harvested in autumn and then stored under dark and naturally cool conditions in a storage house covered with soil.

### Laboratory resistance test using storage root slices

**Fig. 1.**
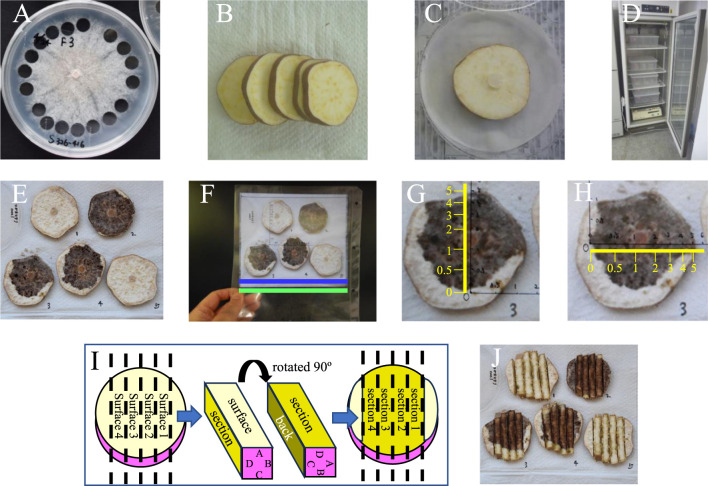
The laboratory testing method using storage root slices. A, Preparation of *D. destruens* blocks. Blocks of an SPDA plate with a diameter of 8.5 mm were cut out from the edge of the spread area of the F3 strain after incubation at 25°C for 2 weeks. B, Preparation of storage root slices. Slices were cut out from the middle part of the root. C, Inoculation of *D. destruens*. The slice was put on a paper filter in a plastic petri dish. F3 was inoculated by putting 1 block of an SPDA plate on the center of the slice. One SPDA block without *D. destruens* was used for the control treatment. D, Incubation. The inoculated and control slices were incubated under dark conditions. E, F, G, H, I, and J, Evaluation of resistance. After incubation, we transferred the slices onto a paper towel and photographed the surface of each using a digital camera (E). The photo was enlarged or reduced on a computer monitor to match the width of the paper towel in the photo (blue line) to the actual width of the paper towel drawn on a handmade scale of a clear plastic sheet placed on the monitor screen (green line) (F). The rotted area index (RAI) was measured with the handmade scale, on which the positions of diameters corresponding to 0, 0.5, 1, 2, 3, 4, 5, 6, 7, 8, 9, and 10 of RAI were marked on the vertical and horizontal axes (yellow lines). RAI ‘1’ equals 1.25 π cm^2^. The RAI of the surface was the average of the vertical and horizontal RAIs (G, H). Four 7-mm-wide sticks were cut out from each slice and rotated 90º to the right so that 4 cross sections of the slice appeared on the top (I). We photographed the cross sections and both ends of the slice, then calculated RAI using the same method to measure the RAI of the slice surface (J). In E and J, slices 1 and 5 were controls without inoculation. Slices 2, 3, and 4 were inoculated with F3. The RAIs of slices 1 and 5 were 0 (the surface, the cross sections, and the adjustments were all 0). The respective RAIs of slices 2, 3, and 4 were 13.0 (the surface, the cross sections, and the adjustments were 5.5, 5.5, and 2, respectively), 11.0 (5, 5, and 1) and 9.0 (4.5, 4.5, and 0). The average RAI of this replication was 11.0.

The laboratory test using sweetpotato storage root slices to measure resistance to foot rot was based on the method of a tuber slice bioassay of potato to confirm the phytotoxicity of thaxtomins and culture extracts of *Streptomyces* strains (Loria *et al.* 1995). F3 was incubated on a sweetpotato dextrose agar (SPDA) plate (Fujiwara *et al.* 2021, Tabuchi *et al.* 2024) at 25°C for 2 weeks. Blocks with an 8.5-mm-diameter SPDA plate were cut out from the edge of the spread area of F3 (Fig. 1A). Several transverse root slices with a thickness of 7 mm were cut out from the middle part of a healthy storage root (Fig. 1B). One storage root slice was placed on a filter paper wetted with 1.5 ml of distilled water in a 9-cm-diameter plastic petri dish, and an SPDA block with F3 was placed inversely on the center of the storage root slice so that F3 directly contacted its surface (Fig. 1C). An SPDA block without *D. destruens* was used for the control treatment. The inoculated and control storage root slices were incubated under dark conditions at 25°C, 30°C, or 35°C for 10 to 15 days (Fig. 1D). To prevent drying, the filter paper was wetted with 1.5 ml of distilled water at 3 and 7 days after inoculation (DAI). After incubation, the slices were transferred onto a paper towel and the surface of each slice was photographed using a digital camera (Fig. 1E). To measure the degree of the rotted area index (RAI), the photo was enlarged or reduced on a computer monitor to match the width of the paper towel in the photo to the actual width drawn on a handmade scale on a clear plastic sheet placed across the computer monitor screen (Fig. 1F). RAI was measured with the handmade scale, where the positions of diameters corresponding to 0, 0.5, 1, 2, 3, 4, 5, 6, 7, 8, 9, and 10 of RAI were marked on the vertical and horizontal axes. We determined the vertical or horizontal RAI as 0, 0.25, 0.5, 0.75, 1, 1.5, 2, 2.5, 3, 3.5, 4, 4.5, 5, 5.5, 6, 6.5, 7, 7.5, 8, 9, or 10 using the handmade scale. RAI ‘1’ equals 1.25 π cm^2^. The surface’s RAI was the average of the vertical and horizontal RAIs of the surface (Fig. 1G, 1H). Next, to investigate how foot rot penetrated the slice interior, 4 sticks with a width of 7 mm were cut out from each storage root slice and rotated 90º to the right so that each cross section of each slice appeared at the top (Fig. 1I). The cross sections and both ends of the slices were then photographed, and the RAIs of the slice surfaces were calculated (Fig. 1J). RAI for 1 slice was calculated by adding the RAIs of the surface, the 4 cross sections, and both ends of the slice. Because accurate RAI could not be calculated when the rotted area reached the edge of the storage root slice, it was adjusted by adding 1 or 2 when rot spread to 30 to 70% or more than 70% of the edge line of the storage root slice, respectively. For 1 cultivar, 3 to 4 slices were usually inoculated, and 1 to 2 slices were used as negative controls. Because there were rarely rotted slices due to causes other than foot rot or uninfected slices among inoculated slices, 1 replication of a test for 1 cultivar consisted of 2 to 4 infected slices cut out from the same storage root. The mean RAI of 2 to 4 infected slices was calculated for 1 replication of a test. We changed storage roots for each replication. The final RAI of each cultivar was determined as the average of all replications. The statical analysis was carried out using JMP (SAS Institute, Cary, NC, USA).

### Examination of various conditions for the laboratory resistance test using storage root slices

We selected 5 cultivars—‘Ayamurasaki’, ‘J-Red’, ‘Kokei No. 14’, ‘Koganesengan’, and ‘Koganemasari’—to examine conditions for the laboratory resistance test using storage root slices, because the resistance levels of these cultivars’ storage roots differed in preliminary tests.

To determine the optimal DAI for the resistance test, the RAIs of storage root slices incubated at 25°C were compared between short-term (10 to 11 days) and long-term (13 to 15 days) incubation after inoculation. To determine the optimal temperature during incubation, RAI after short-term incubation was compared at 25°C, 30°C, and 35°C.

### Correlation analysis

We calculated the correlation between the storage period of storage roots and RAI. The storage period is the number of days from the harvesting of storage roots to the inoculation of *D. destruens* into storage root slices (Supplemental Fig. 1).

The difference in the average storage period of storage roots between cultivars was investigated using a *t*-test.

### Field resistance test to foot rot

Field resistance testing methods for foot rot were described by Kobayashi *et al.* (2025) and Kyushu Okinawa Agricultural Research Center, NARO (2023). The resistances of 15, 20, and 12 cultivars in 2020, 2021, and 2022, respectively, were field tested. ‘Amahazuki’ was field tested in 2022, but the resistance evaluation of ‘Amahazuki’ was not shown because RAI was not investigated. These tests were conducted in 3 plots (plots A, B, and C), with 30 cuttings per plot in 2020 and 2021 and 24 cuttings per plot in 2022 for each cultivar (Tables 3–5, Supplemental Table 1). Healthy stem cuttings disinfected with benomyl were planted in fields naturally infested with *D. destruens* on May 9, 6, and 10, and storage roots were harvested on October 6, 13, and 18, in 2020, 2021, and 2022, respectively. The proportions of the plants rotted at the basal part of stem (PRS) of each cultivar were investigated on July 13, July 29, August 11, August 24, September 11, September 25, and October 6 in 2020; July 5, July 19, August 3, August 20, September 2, September 19, and October 12 in 2021; and August 23, September 20, October 7, and October 17 in 2022. The yields and the numbers of storage roots with a rot index of 0 to 5 of each cultivar were investigated at harvest. The rot indices of storage roots in 2020 and 2021 were as follows: 0, no symptom; 1, germination; 2, rotted only in the root stalk; 3, less than half rotted; 4, more than half rotted; 5, rotted wholly (Kobayashi *et al.* 2025). The rot indices of storage roots in 2022 were as follows: 0, no symptom; 1, germination; 2, rotted only in the root stalk or less than half rotted; 4, more than half rotted; 5, rotted wholly. The rot index 2 in 2022 included the rot index 3 (less than half rotted) in 2020 and 2021. Because the infected storage roots tended to germinate even when no symptom of foot rot was observed, we investigated germinated storage roots. However, it should be noted that uninfected storage roots of some cultivars, e.g., ‘Benimasari’ in this study, germinated more frequently than other cultivars in fields (Ishiguro *et al.* 2004). PRS of cultivars and the yields of storage roots with each rot index were reported by Kobayashi *et al.* (2025) and Kyushu Okinawa Agricultural Research Center, NARO (2023). The degree of severity of foot rot in storage roots was calculated based on the sum of the products of each rot index and the number of storage roots with each rot index. Resistance to foot rot among the cultivars in the field test was determined comprehensively by considering the degree of severity of foot rot in storage roots, the proportion of infected storage root weight, and the total yield of storage roots, with emphasis on PRS. The resistance evaluation categories from high to low resistance in the field tests were as follows: R, resistant; Rʹ, less resistant; I, intermediate; Sʹ, less susceptible; S, susceptible. The resistance evaluation in the field test was based on Kyushu Okinawa Agricultural Research Center, NARO (2023) (Table 2).

The correlations between RAI and the 7 categories in the field test (R; Rʹ; I; I or Sʹ; Sʹ; S or Sʹ; and S) or PRS in the field test were calculated. The categories were represented numerically as follows: R = 1, Rʹ = 2, I = 3, I or Sʹ = 3.5, Sʹ = 4, S or Sʹ = 4.5, and S = 5 (Table 2).

### Proportional analysis of rot indices in storage roots

Foot rot appears to invade storage roots through the basal stems in fields (Kobayashi 2022). When the proportion of infected stems relative to all stems of a cultivar is low, likely due to stem resistance, the proportion of infected storage roots among all harvested roots of the cultivar also tends to be low, even if the cultivar’s storage roots are susceptible to foot rot. Therefore, the resistance of a storage root itself can’t be precisely evaluated according to the proportion of infected storage roots in field tests. The resistance of a storage root should be evaluated by how the symptom of foot rot advanced after invading the storage roots. We thought we could evaluate a cultivar’s storage root resistance by investigating the proportion of severely rotted storage roots. This is defined as the proportion of storage roots with severe symptoms of foot rot (e.g., indices 3, 4, and 5) relative to the total number of storage roots with both less severe and more severe symptoms (e.g., indices 2, 3, 4, and 5). Therefore, we analyzed the proportion of storage roots with rot indices of 1 or higher to those with more advanced rot indices, based on the number of storage roots with each rot index for each cultivar (Tables 3–5, Supplemental Table 1). For example, to calculate the proportion of storage roots with rot indices 3, 4, and 5 to those with indices 2, 3, 4, and 5 (represented as 3–5/2–5) for ‘Murasakimasari’ in 2020, we used the following method. In plot A, the total number of storage roots with rot indices 3–5 was 22, which was the sum of 14 (index 3), 3 (index 4), and 5 (index 5). The total number of storage roots with rot indices 2–5 in plot A was 56, calculated as the sum of 34 (index 2), 14 (index 3), 3 (index 4), and 5 (index 5). The proportion was 39.3% (22/56) in plot A. The same calculation was done in plots B and C, resulting in 27.3% and 46.2% proportions, respectively. Finally, the mean proportion for the 3 plots was calculated as 37.6% for the proportion of 3–5/2–5 of ‘Murasakimasari’ in 2020.

In 2021, data from plot B of ‘Murasakimasari’, plot A of ‘Beniotome’, plot C of ‘Konahomare’, and plot C of ‘Michishizuku’ were excluded due to severe field conditions caused by poor drainage.

## Results

### Optimizing incubation terms and temperatures for resistance testing

In our examination of optimal incubation terms in the laboratory test, the RAI of long-term incubation was larger than those of short-term incubation in ‘Koganesengan’ and the mean of 5 cultivars (Supplemental Table 2). We chose short-term incubation for the storage root test because the rotted areas of storage root slices in ‘Koganesengan’ and ‘Koganemasari’ often reached the edges during long-term incubation, making it difficult to calculate RAI accurately.

Next, RAIs after short-term incubation at 25°C, 30°C, and 35°C were compared (Supplemental Table 3). In almost all cultivars, the rotted areas of slices incubated at 35°C did not spread. RAI after incubation at 30°C was greater than that at 25°C. These results agreed with the observations that *D. destruens* spread on SPDA plates faster in the order of 30°C, 25°C, and 35°C (Nishioka *et al.* 2021). The rotted areas of ‘Koganesengan’ and ‘Koganemasari’ incubated at 30°C often reached the slice edge, making it difficult to calculate RAI accurately. We chose 25°C for the storage root test.

Based on these results, the laboratory resistance test of storage roots was carried out as follows: slices inoculated with *D. destruens* were incubated at 25°C, and RAI was measured on 10 or 11 DAI.

### Resistance of storage roots under laboratory conditions

We investigated the foot rot resistance of storage roots of 46 sweetpotato cultivars (Table 2). Their RAIs ranged from 2.8 to 10.6. A positive correlation was detected between the storage period and RAI in ‘Ayamurasaki’, ‘Konahomare’, ‘Churakoibeni’, and ‘Michishizuku’ (Supplemental Fig. 1). A negative correlation was detected between the storage period and RAI in ‘Suzuhokkuri’, ‘Murasakihomare’, ‘Beniotome’, and ‘Ayakomachi’ (Supplemental Fig. 1).

RAI was compared among 46 cultivars by the Kruskal-Wallis test, and a significant difference was detected (*p* < 0.05).

### Correlation between RAI in the laboratory test and the rot index of storage roots in the field test

Storage root resistance to foot rot can be evaluated by RAI in a laboratory test without the influence of stem resistance. We thought we could evaluate storage root resistance by investigating the proportion of severely rotted storage roots in the field test as described in Materials and Methods, too. Therefore, we analyzed the correlation between RAI in the laboratory test and the proportion of storage roots with rot indices of 1 or higher to the number of storage roots with more advanced rot indices in the field test based on data from Kobayashi *et al.* (2025) and Kyushu Okinawa Agricultural Research Center, NARO (2023) (Supplemental Table 1). First, we calculated the proportion of storage roots at each rot index to the total number of all harvested storage roots of 15, 20, and 11 cultivars in the field tests in 2020, 2021, and 2022, respectively. No significant correlation was detected between RAI and the proportion of roots with a rot index of 0 relative to the total number of roots across all rot indices (0–5), represented as 0/0–5, in these 3 years (data not shown). No significant correlation was detected between RAI and any other proportion represented as 1/0–5, 2/0–5, 3/0–5, 4/0–5, or 5/0–5 in those 3 years (data not shown). Next, we calculated the proportion of roots with a rot index of 1 or higher to those with more advanced rot indices. A significant correlation was observed between RAI and the proportion of 3–5/2–5 for 15 cultivars in 2020 (Table 3) and 4–5/3–5 for 20 cultivars in 2021 (Table 4). However, no significant correlation was observed in 2022 (Table 5).

We also analyzed the correlation between RAI in the laboratory test and PRS in the field tests based on the data from Kobayashi *et al.* (2025) and Kyushu Okinawa Agricultural Research Center, NARO (2023). No significant correlation was observed (Supplemental Table 4).

## Discussion

### Resistance index of storage roots

Usui and Kushima (2020) also reported a method of evaluating storage root resistance to foot rot by indexing the proportion of rotted area of a root slice. However, this index seemed to be unsuitable. Even if the absolute rotted area of a slice is the same, the proportion of rotted area differs depending on the area of the storage root slice, e.g., the proportion of rotted area varies depending on the slice size. For example, the proportion is small when slice is large, whereas it is large when the slice is small. Therefore, we used the rotted area itself as an index.

### Correlation between the storage period and RAI

Positive correlations were detected between the storage period of storage roots and RAI in ‘Ayamurasaki’, ‘Konahomare’, ‘Churakoibeni’, and ‘Michishizuku’. On the other hand, negative correlations were detected in ‘Suzuhokkuri’, ‘Murasakihomare’, ‘Beniotome’, and ‘Ayakomachi’ (Table 2, Supplemental Fig. 1). This is unexplained and very interesting. It was reported that the contents of several substances, such as sugars, starches, water, β-carotene, and anthocyanin, changed during root storage, and the states of change among sugars, starches, water, and β-carotene varied depending on the cultivar (Ando *et al.* 2018, Takahata *et al.* 1995, Watanabe *et al.* 1999). Some unidentified substances or gene products may be related to the resistance of storage roots, and their states of change may vary depending on the cultivar. We did not analyze how substances or gene products changed in the storage roots in this study. Further study is needed.

We had to take care to ensure that the timing at which we investigated the RAI of these 8 cultivars, where significant correlations were detected between the storage period of the roots and RAI, was not biased. The average root storage period in the laboratory tests of ‘Ayamurasaki’, ‘Konahomare’, ‘Churakoibeni’, ‘Michishizuku’, ‘Suzuhokkuri’, ‘Murasakihomare’, ‘Beniotome’, and ‘Ayakomachi’ were 192.5, 205.9, 204.3, 153.5, 142.1, 206.2, 131.6, and 131.6 days, respectively. The average for the other 38 cultivars was 185.8 days, not significantly different from those of ‘Ayamurasaki’, ‘Konahomare’, ‘Churakoibeni’, and ‘Murasakihomare’ but significantly longer than those of ‘Michishizuku’ (*p* < 0.05), ‘Suzuhokkuri’ (*p* < 0.01), ‘Beniotome’ (*p* < 0.01), and ‘Ayakomachi’ (*p* < 0.01). The root storage periods of ‘Michishizuku’, ‘Suzuhokkuri’, ‘Beniotome’, and ‘Ayakomachi’ were shorter than the average of the 38 cultivars. A positive correlation was found for ‘Michishizuku’, whereas ‘Suzuhokkuri’, ‘Beniotome’, and ‘Ayakomachi’ exhibited negative correlations between the root storage periods and RAI. If the average storage period of these 4 cultivars did not significantly differ from that of the 38 cultivars, RAI might be higher in ‘Michishizuku’ or lower in ‘Suzuhokkuri’, ‘Beniotome’, and ‘Ayakomachi’ compared to our results (9.2, 3.6, 5.2, and 8.0, respectively) (Table 2). On the other hand, no significant correlation was detected between the storage period and RAI in the 38 cultivars other than the 8 described above (Table 2, Supplemental Fig. 1). In these 38 cultivars, the laboratory resistance tests could be carried out regardless of the storage period.

### Variation in resistance of storage roots among cultivars

We investigated the resistance of storage roots of 46 cultivars. Significant differences among cultivars were observed for the resistance of storage roots (Table 2). Unfortunately, we have not yet found a fully resistant cultivar in which RAI was zero. Therefore, we can evaluate only the ‘relative’ foot rot resistance of storage roots.

We classified the resistance of cultivar storage roots into 3 groups based on RAI: resistant (RAI < 5.5), intermediate (RAI 5.5–8.0), and susceptible (RAI > 8.0), based on the segregation of histograms of 46 cultivars (Supplemental Fig. 2).

### Correlation between resistance indices in laboratory tests and proportions of rot indices of storage roots in field tests

Significant correlations were observed between RAI in the laboratory test and the proportions of 3–5/2–5 in 2020 and 4–5/3–5 in 2021 in the field test (Tables 3, 4, Supplemental Fig. 3), which were the proportions of severely rotted storage roots in the field test. On the other hand, in 2020, 2021, and 2022, no significant correlation was detected between RAI in the laboratory test and the proportion of 1–5/0–5 or 2–5/0–5, which was the proportion of unhealthy storage roots and that of infected storage roots in the field test, respectively. Moreover, on all investigated days in all 3 years, no significant correlation was observed between RAI in the laboratory test and PRS in the field test, an index of stem resistance (Supplemental Table 4). And no significant correlation was detected between the resistance evaluations in the field tests of 21 cultivars and RAI in the laboratory test (*p* = 0.20) (Table 2). These results indicated that RAI in the laboratory test can’t be used as an index to estimate indirectly the proportion of unhealthy storage roots, the proportion of infected storage roots, the stem resistance, or the resistance evaluations in field tests. However, RAI may be used as such an index of the proportion of severely rotted storage roots in field tests in some cases, resulting that the laboratory storage roots test can indirectly evaluate the foot rot resistance of storage roots in the field test.

No significant correlation was observed between RAI in the laboratory test and the proportion of severely rotted storage roots in the field test in 2022 (Table 5). The proportion of the mean of 2–5/0–5, 4–5/0–5, and 5/0–5 in 2022 was lower than those in 2020 and 2021, indicating that foot rot damage to storage roots in fields was less severe in 2022 than in 2020 or 2021 (Tables 3–5). This low degree of damage and lower number of tested cultivars could explain why a significant correlation was not detected in 2022. Furthermore, accurate evaluation of storage root resistance among various sweetpotato cultivars in the field tests seemed very difficult because fungal density was not uniform in the fields, and the period from foot rot infection in storage roots to harvesting and investigation differed from root to root even within the same individual plant, among plants within the same cultivar, and among cultivars. This could be the reason why no significant correlation was detected between RAI and the proportion excluding 3–5/2–5 in 2020 and 4–5/3–5 in 2021 in the field test.

In this study, we improved the laboratory test to measure the foot rot resistance of storage roots, and previously we developed a laboratory test to measure foot rot resistance of stems (Tabuchi *et al.* 2024). The former and the latter can indirectly evaluate the foot rot resistance of storage roots and stems, respectively, in the field test which is usually used to evaluate cultivar resistance in breeding programs. We can investigate the resistance of stems and storage roots of sweetpotato cultivars in a laboratory year-round without any effects from environmental factors which often cause problems in the field test. These laboratory resistance tests of stems and storage roots can help breed resistant cultivars.

## Author Contribution Statement

HT conducted the laboratory tests, analyzed the data, and drafted the manuscript. YOK, AK, and HT improved the laboratory resistance test to foot rot using storage roots. HT and AK prepared storage roots of sweetpotato for laboratory tests. YOK contributed to the preparation of *D. destruens*. AK conducted the fields test. AK, YK, TS, and KS examined the field tests. All authors contributed to the manuscript and approved the submitted version.

## Supplementary Material

Supplemental Figures

Supplemental Tables

## Figures and Tables

**Table 1.** Sweetpotato cultivars used in this study

Cultivar	Usage	Share (%)*^a^*		Cultivar	Usage	Share (%)*^a^*
Beniazuma	Table use	9.2		Akemurasaki	Processed foods	NA
Beniharuka	Table use	21.9		Ayakomachi	Processed foods	NA
Benihinata*^b^*	Table use	NA		Ayamurasaki	Processed foods	0.2
Benimasari	Table use	4.4		Benihayato	Processed foods	NA
Beniotome	Table use	<0.1		Churakanasa	Processed foods	NA
Fukumurasaki	Table use	NA		Churakoibeni	Processed foods	0.5
Kokei No. 14	Table use	8.2		Hamakomachi	Processed foods	NA
Kyushu No. 138	Table use	NA		Joy White	Processed foods	<0.1
Norin No. 1	Table use	<0.1		J-Red	Processed foods	NA
Purple Sweet Lord	Table use	0.1		Koganemasari	Processed foods	NA
Suzuhokkuri	Table use	NA		Kyushu No. 137	Processed foods	NA
Daichinoyume	Raw materials	<0.1		Murasakihomare	Processed foods	NA
Koganesengan	Raw materials	20.5		Murasakimasari	Processed foods	0.6
Konahomare	Raw materials	<0.1		Okikogane	Processed foods	NA
Konaishin	Raw materials	5.1		Origin Ruby	Processed foods	NA
Konamizuki	Raw materials	NA		Satsumaakane	Processed foods	NA
Michishizuku	Raw materials	NA		Satsumahikari	Processed foods	NA
Satsumamasari	Raw materials	NA		Sunny Red	Processed foods	NA
Norin No. 2	Raw materials	0.1		Suzukogane	Processed foods	NA
Shiroyutaka	Raw materials	4.9		Tamaakane	Processed foods	<0.1
95K1	Genetic resources	NA		Tamaotome	Processed foods	<0.1
Choshu	Genetic resources	NA		Tamayutaka	Processed foods	0.2
Miyano No. 7	Genetic resources	NA		Tokimasari	Processed foods	NA

*^a^* The production area share of each cultivar is based on data from the Ministry of Agriculture, Forestry and Fisheries (2024). NA indicates that there are no data on production area shares of these cultivars. *^b^* ‘Benihinata’ is described as ‘Kyushu No. 201’ at Kyushu Okinawa Agricultural Research Center, NARO (2023).

**Table 2.** Rotted area index (RAI) in laboratory tests and resistance evaluation in field tests of 46 cultivars

Cultivar	Laboratory test	Field test		Cultivar	Laboratory test	Field test
	RAI*^a^* ± *SE* (replication)	Resistance evaluation*^b^*			RAI*^a^* ± *SE* (replication)	Resistance evaluation*^b^*
Satsumahikari	2.8 ± 0.3 (23)			Norin No. 1	7.0 ± 0.7 (13)	
Murasakimasari	3.0 ± 0.4 (33)	I		Churakanasa	7.0 ± 0.6 (13)	
Benihayato	3.2 ± 0.3 (19)	R		Hamakomachi	7.1 ± 0.6 (30)	
Ayamurasaki*^c^*	3.2 ± 0.3 (33)	I		Daichinoyume	7.2 ± 0.8 (14)	S
Suzuhokkuri*^d^*	3.6 ± 0.3 (24)	Rʹ		Fukumurasaki	7.2 ± 0.5 (25)	
Benimasari	3.7 ± 0.6 (13)	Rʹ		Kokei No. 14	7.3 ± 0.5 (32)	Sʹ
Akemurasaki	3.8 ± 0.6 (13)			Konahomare*^c^*	7.5 ± 0.6 (21)	S
Suzukogane	3.9 ± 0.6 (14)			Kyushu No. 138	7.7 ± 0.8 (15)	
Origin Ruby	3.9 ± 0.3 (22)			Tamayutaka	7.9 ± 0.9 (13)	I
Okikogane	4.0 ± 0.3 (20)	R		Ayakomachi*^d^*	8.0 ± 0.5 (11)	
J-Red	4.4 ± 0.5 (21)			Churakoibeni*^c^*	8.1 ± 0.6 (25)	
Murasakihomare*^d^*	4.5 ± 0.6 (19)			Satsumamasari	8.7 ± 0.6 (26)	I
Benihinata*^e^*	4.7 ± 0.4 (13)	R		Kyushu No. 137	8.7 ± 0.7 (15)	
Beniotome*^d^*	5.2 ± 0.5 (14)	I		Tokimasari	8.8 ± 0.9 (13)	
Sunny Red	5.6 ± 0.6 (15)			Shiroyutaka	8.9 ± 0.7 (18)	I or Sʹ
Miyano No. 7	5.6 ± 0.8 (14)			Satsumaakane	9.0 ± 0.7 (26)	
Choshu	5.8 ± 0.6 (16)			Tamaakane	9.0 ± 0.7 (18)	R
Konamizuki	6.1 ± 0.6 (29)	S		Koganemasari	9.2 ± 0.3 (41)	
Beniazuma	6.1 ± 0.6 (30)	Sv		Michishizuku*^c^*	9.2 ± 0.7 (14)	Rʹ
Tamaotome	6.3 ± 0.8 (13)			Joy White	9.8 ± 0.4 (16)	
Konaishin	6.6 ± 0.7 (34)	Rʹ		95K1	10.0 ± 0.7 (14)	
Beniharuka	6.6 ± 0.5 (32)	S or Sʹ		Norin No. 2	10.1 ± 0.7 (13)	
Koganesengan	6.7 ± 0.5 (33)	Sʹ		Purple Sweet Lord	10.6 ± 0.8 (13)	

*^a^* RAI was compared among 46 cultivars by the Kruskal-Wallis test, and a significant difference was detected (*p* < 0.05). *^b^* The resistance evaluation to foot rot in field tests was based on Kyushu Okinawa Agricultural Research Center, NARO (2023). The resistance evaluation categories from high to low resistance in the field tests were as follows: R, resistant; Rʹ, less resistant; I, intermediate; Sʹ, less susceptible; S, susceptible. No significant correlation was detected between the resistance evaluations and RAI of 21 cultivars (*p* = 0.20). *^c^* A positive correlation was detected between the storage period and RAI (*p* < 0.05). *^d^* A negative correlation was detected between the storage period and RAI (*p* < 0.05). *^e^* ‘Benihinata’ is described as ‘Kyushu No. 201’ at Kyushu Okinawa Agricultural Research Center, NARO (2023).

**Table 3.** Correlation between proportions of rot indices of storage roots in field tests in 2020 and RAI in laboratory tests

Cultivar	Laboratory test		Field test in 2020														
	RAI		Proportion of rot index of storage roots*^a^* (%)														
			1–5 /0–5	2–5 /0–5	3–5 /0–5	4–5 /0–5	5 /0–5	2–5 /1–5	3–5 /1–5	4–5 /1–5	5 /1–5	3–5 /2–5	4–5 /2–5	5 /2–5	4–5 /3–5	5 /3–5	5 /4–5
Murasakimasari	3.0		38.8	27.7	10.3	5.4	3.1	70.7	26.7	15.1	9.3	37.6	21.6	13.2	54.7	29.8	47.5
Ayamurasaki	3.2		14.2	6.5	1.8	0.5	0.5	48.6	15.6	3.0	3.0	26.2	4.8	4.8	25.0	25.0	100.0
Benimasari	3.7		49.2	4.8	2.6	1.3	0.0	10.1	5.4	2.7	0.0	43.3	21.7	0.0	50.0	0.0	0.0
Konamizuki	6.1		57.3	42.7	30.5	5.6	2.2	72.7	52.2	9.5	4.1	72.1	13.0	5.9	18.3	8.4	45.6
Beniazuma	6.1		30.2	24.3	14.6	4.0	1.7	80.2	49.9	12.2	5.4	62.3	14.8	6.6	24.3	10.5	41.7
Beniharuka	6.6		75.7	28.5	18.5	6.3	4.3	37.4	25.0	8.5	5.8	68.6	23.8	16.4	34.0	22.7	77.8
Konaishin	6.6		7.5	7.5	6.0	1.7	0.4	100.0	70.7	22.9	5.1	70.7	22.9	5.1	35.8	5.6	13.3
Koganesengan	6.7		42.0	24.5	15.0	6.8	1.4	58.8	34.5	15.4	3.3	61.2	27.7	5.1	43.8	10.4	25.4
Daichinoyume	7.2		81.7	73.0	59.7	28.3	5.4	89.1	72.6	34.6	6.6	81.4	38.8	7.4	48.5	9.1	19.2
Kokei No. 14	7.3		66.8	42.1	36.2	24.9	7.5	65.0	55.3	37.8	11.3	86.2	58.3	16.1	67.5	18.6	25.2
Konahomare	7.5		54.4	53.1	47.1	31.7	14.9	97.4	86.7	58.8	28.3	89.0	60.6	29.3	68.3	32.9	46.3
Tamayutaka	7.9		23.0	5.6	2.8	0.9	0.0	23.3	10.9	3.6	0.0	38.9	13.9	0.0	37.5	0.0	0.0
Satsumamasari	8.7		33.2	31.6	24.9	10.5	5.7	93.7	74.8	31.2	15.9	79.9	33.0	16.7	40.2	20.3	37.5
Shiroyutaka	8.9		44.3	25.9	20.1	7.6	4.3	59.3	43.6	14.9	8.4	74.7	26.3	14.8	30.3	17.0	56.1
Tamaakane	9.0		3.3	1.3	0.6	0.2	0.2	41.4	15.0	7.3	7.3	48.3	18.3	18.3	62.5	62.5	100.0
Mean	6.6		41.4	26.6	19.4	9.0	3.4	63.2	42.6	18.5	7.6	62.7	26.6	10.6	42.7	18.2	42.4
Correlation coefficient*^b^*																	
RAI			0.01	0.19	0.30	0.28	0.29	0.22	0.39	0.35	0.33	0.60*	0.37	0.42	0.16	0.21	–0.01

*^a^* Rot index was as follows: 0, no symptom; 1, germination; 2, rotten only in root stalk; 3, less than half rotted; 4, more than half rotted; 5, rotted wholly. For example, 1–5/0–5 means the proportion of the total number of storage roots with rot indices 1–5 to the total number of storage roots with rot indices 0–5. *^b^* Correlation coefficients were calculated between RAI in the laboratory tests and the proportion of rot index of storage roots in the field tests. * *p* < 0.05.

**Table 4.** Correlation between proportions of rot indices of storage roots in field tests in 2021 and RIA in laboratory tests

Cultivar	Laboratory test		Field test in 2021														
	RAI		Proportion of rot index of storage roots*^a^* (%)														
			1–5 /0–5	2–5 /0–5	3–5 /0–5	4–5 /0–5	5 /0–5	2–5 /1–5	3–5 /1–5	4–5 /1–5	5 /1–5	3–5 /2–5	4–5 /2–5	5 /2–5	4–5 /3–5	5 /3–5	5 /4–5
Murasakimasari	3.0		70.9	52.1	46.0	12.9	6.2	75.3	66.6	19.0	8.9	88.1	24.5	11.9	27.7	13.5	49.8
Ayamurasaki	3.2		38.1	29.2	17.5	1.6	0.8	72.5	38.3	4.0	2.0	48.0	5.0	2.5	9.0	4.5	50.0
Benihayato	3.2		10.0	8.3	6.3	2.5	1.6	74.0	52.6	17.4	12.6	75.9	27.8	22.8	31.0	24.6	77.8
Suzuhokkuri	3.6		39.1	30.0	20.8	5.9	4.7	77.0	52.6	14.8	12.1	68.5	19.4	15.7	28.1	23.3	83.3
Benimasari	3.7		47.9	26.3	22.2	5.0	1.5	54.9	46.5	10.4	3.5	82.4	18.4	6.8	22.2	9.1	42.2
Okikogane	4.0		9.4	5.9	5.5	2.0	0.8	68.2	62.6	22.7	8.6	93.3	32.8	13.9	36.1	13.9	50.0
Beniotome	5.2		52.4	47.0	28.8	5.6	2.6	89.6	54.9	10.7	4.9	61.0	11.9	5.5	19.9	8.8	46.7
Konamizuki	6.1		84.5	77.7	66.6	27.3	10.8	92.0	78.7	32.5	12.8	86.2	36.0	14.4	41.5	16.2	39.9
Beniazuma	6.1		73.2	63.3	44.3	14.1	7.3	86.4	60.3	19.2	9.9	69.5	22.1	11.5	31.8	16.4	51.6
Beniharuka	6.6		91.5	58.5	46.2	16.9	9.8	64.1	49.7	18.3	10.7	78.5	28.8	16.6	37.0	22.3	59.6
Konaishin	6.6		32.3	30.5	17.2	7.4	3.9	94.0	51.8	21.6	10.9	54.9	22.8	11.4	41.8	20.2	45.0
Koganesengan	6.7		83.0	68.2	61.7	36.1	13.4	82.3	74.6	43.3	16.4	90.5	52.7	19.7	58.5	21.6	37.9
Daichinoyume	7.2		84.8	81.6	72.4	47.4	27.7	96.2	84.9	54.9	32.7	88.2	56.9	34.0	63.9	38.6	63.1
Kokei No. 14	7.3		86.8	74.2	65.7	25.4	13.5	85.3	75.1	29.0	15.1	87.9	33.4	17.1	38.0	19.1	48.5
Konahomare	7.5		90.7	87.2	78.4	55.1	29.0	96.2	86.4	60.7	32.0	89.8	63.1	33.3	70.3	37.2	52.9
Tamayutaka	7.9		45.2	27.6	18.8	3.1	0.3	63.3	44.9	8.9	0.5	69.0	12.0	0.8	15.8	1.2	16.7
Satsumamasari	8.7		68.0	60.3	52.2	32.0	22.7	88.5	76.9	47.2	33.2	87.3	53.3	37.5	61.4	43.4	71.0
Shiroyutaka	8.9		32.7	27.8	19.9	7.4	4.1	87.1	58.1	22.6	11.4	68.8	26.4	14.1	41.4	19.2	49.2
Tamaakane	9.0		7.8	4.9	2.7	1.1	0.8	62.0	35.8	15.9	12.5	56.7	26.1	21.1	46.5	38.4	77.8
Michishizuku	9.2		42.4	36.6	21.0	7.9	2.4	85.9	48.5	17.6	5.8	56.1	20.2	6.8	34.6	12.7	43.3
Mean	6.2		54.5	44.9	35.7	15.8	8.2	79.7	60.0	24.5	12.8	75.0	29.7	15.9	37.8	20.2	52.8
Correlation coefficient*^b^*																	
RAI			0.18	0.25	0.23	0.33	0.34	0.33	0.17	0.39	0.37	–0.09	0.40	0.31	0.54*	0.41	–0.13

*^a^* Rot index was described in Table 3. *^b^* Correlation coefficients were calculated between RAI in the laboratory tests and the proportion of rot index of storage roots in the field tests. * *p* < 0.05.

**Table 5.** Correlation between proportions of rot indices of storage roots in field tests in 2022 and RIA in laboratory tests

Cultivar	Laboratory test	Field test in 2022									
	RAI	Proportion of rot index of storage roots*^a^*									
		1–5/0–5	2–5/0–5	4–5/0–5	5/0–5	2–5/1–5	4–5/1–5	5/1–5	4–5/2–5	5/2–5	5/4–5
Ayamurasaki	3.2	13.0	13.0	2.0	1.2	100.0	18.3	9.7	18.3	9.7	50.0
Benimasari	3.7	51.7	11.3	3.5	0.5	20.8	6.7	1.1	39.6	11.1	16.7
Benihinata*^b^*	4.7	1.1	0.7	0.0	0.0	75.0	0.0	0.0	0.0	0.0	NA
Beniharuka	6.6	80.5	40.7	9.5	3.5	51.4	12.4	4.5	20.8	8.2	38.3
Konaishin	6.6	7.0	6.5	3.1	0.2	94.7	34.0	2.6	36.9	2.8	5.0
Koganesengan	6.7	43.3	31.9	12.1	4.6	73.6	28.5	11.2	38.2	15.5	37.5
Daichinoyume	7.2	65.1	42.4	11.9	1.5	65.0	18.3	2.2	28.7	3.4	13.0
Kokei No. 14	7.3	55.6	33.6	11.1	4.4	60.3	20.1	8.1	34.0	14.1	39.3
Shiroyutaka	8.9	45.5	27.4	6.4	1.7	57.5	12.9	3.2	22.2	5.3	19.4
Tamaakane	9.0	2.6	1.9	0.2	0.0	81.7	33.3	0.0	33.3	0.0	0.0
Michishizuku	9.2	19.3	14.6	4.9	0.2	76.5	25.2	0.9	34.7	1.3	4.5
Mean	6.6	33.1	19.4	5.5	1.5	67.2	17.9	3.6	26.3	5.9	20.3
Correlation coefficient*^c^*											
RAI		0.05	0.22	0.26	0.07	0.09	0.51	–0.25	0.31	–0.34	–0.54

NA, not available. *^a^* Rot index was as follows: 0, no symptom; 1, germination; 2, rotted only in root stalk or less than half rotted; 4, more than half rotted; 5, rotted wholly. Rot index 2 in 2022 included rot index 3 (less than half rotted) in 2020 and 2021. *^b^* ‘Benihinata’ is described as ‘Kyushu No. 201’ at Kyushu Okinawa Agricultural Research Center, NARO (2023). The proportion of 5/4–5 could not be calculated because there were no storage roots with indices 4 and 5. *^c^* No significant correlation coefficients were detected between RAI in the laboratory tests and the proportion of rot index of storage roots in the field tests.
