# Supplementary material for: Resistance of storage roots of 46 sweetpotato cultivars to foot rot disease caused by Diaporthe destruens was evaluated using a laboratory test
Source: Breed Sci. 2025 Aug 20;75(4):315–24. doi: 10.1270/jsbbs.25019 (PMC13051632; doi:10.1270/jsbbs.25019)
Supplement: Supplementary file 1 — Supplemental Figures [file 75_315_s1.pdf]

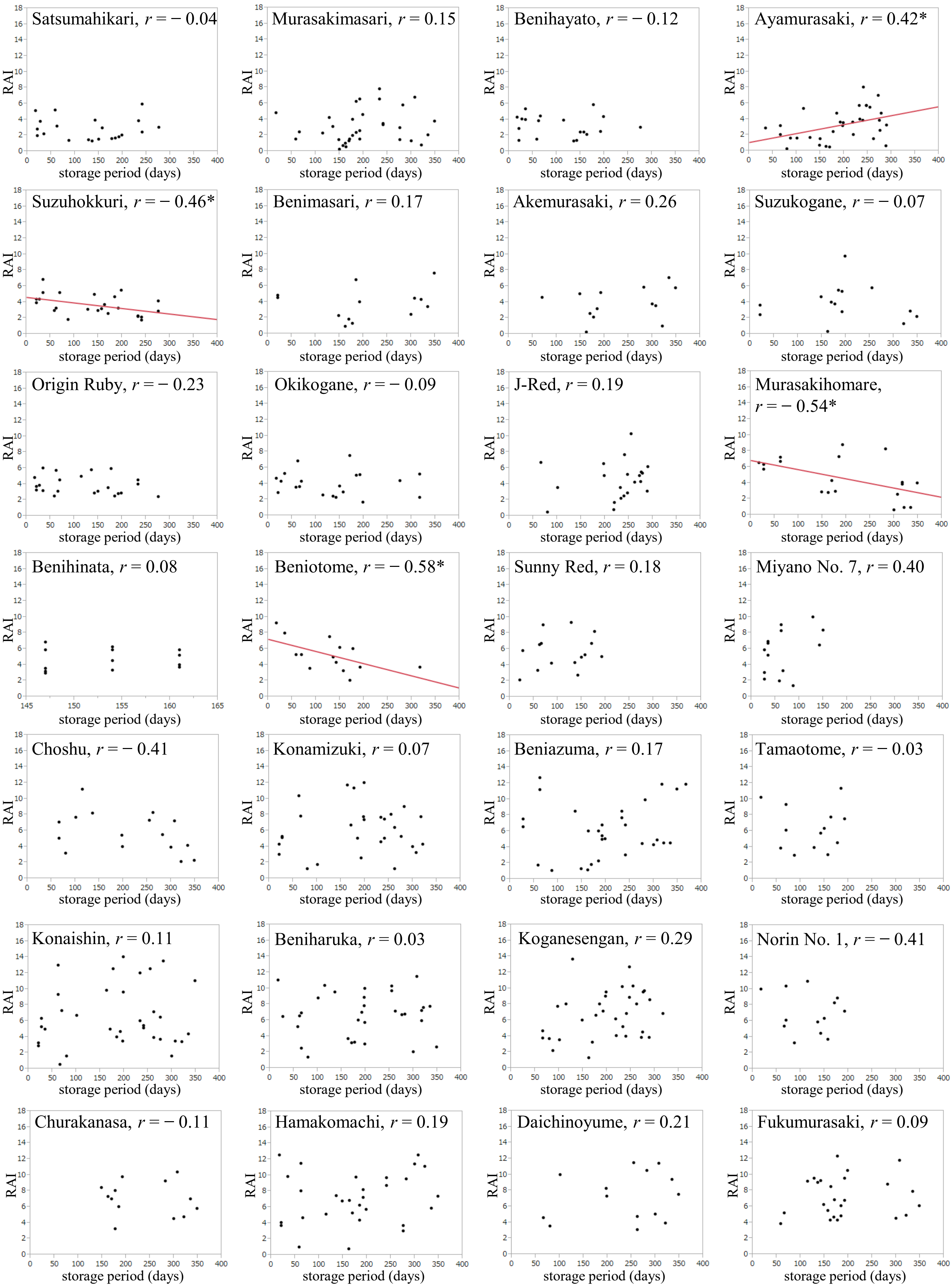
Satsumahikari, r = − 0.04
Murasakimasari, r = 0.15
Benihayato, r = − 0.12
Ayamurasaki, r = 0.42*
Suzuhokkuri, r = − 0.46*
Benimasari, r = 0.17
Akemurasaki, r = 0.26
Suzukogane, r = − 0.07
Origin Ruby, r = − 0.23
Okikogane, r = − 0.09
J-Red, r = 0.19
Murasakihomare, r = − 0.54*
Benihinata, r = 0.08
Beniotome, r = − 0.58*
Sunny Red, r = 0.18
Miyano No. 7, r = 0.40
Choshu, r = − 0.41
Konamizuki, r = 0.07
Beniazuma, r = 0.17
Tamaotome, r = − 0.03
Konaishin, r = 0.11
Beniharuka, r = 0.03
Koganesengan, r = 0.29
Norin No. 1, r = − 0.41
Churakanasa, r = − 0.11
Hamakomachi, r = 0.19
Daichinoyume, r = 0.21
Fukumurasaki, r = 0.09
RAI
storage period (days)

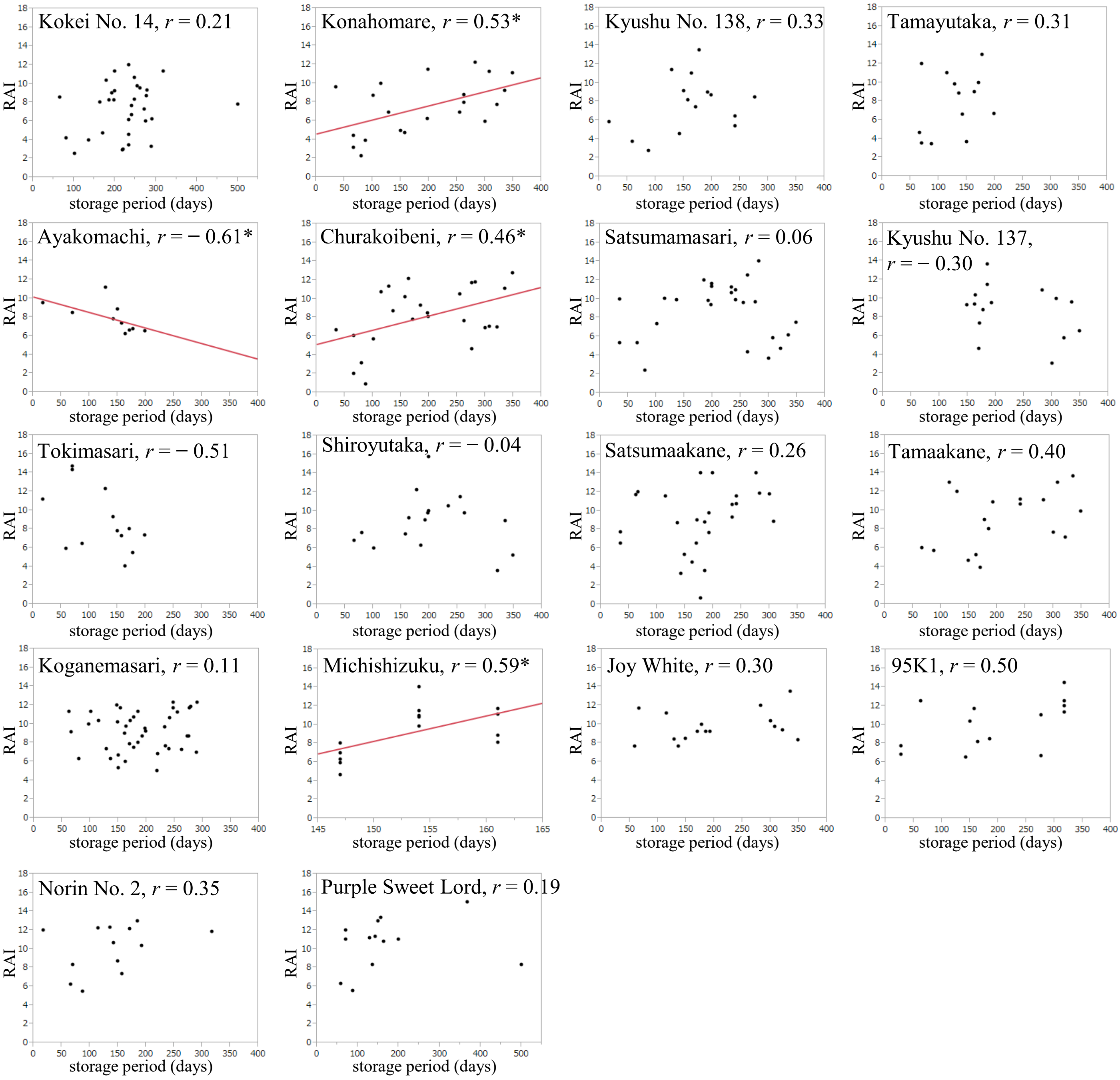

Supplemental Fig. 1 The relationship between the storage period and RAI. For storage period, 0–400 days are indicated for all cultivars except ‘Benihinata’ (145–165), ‘Kokei No. 14’ (0–550), ‘Michishizuku’ (145–165), and ‘Purple Sweet Lord’ (0–550). A regression line is shown when a significant correlation was detected between the storage period and RAI. * $p < 0.05$.

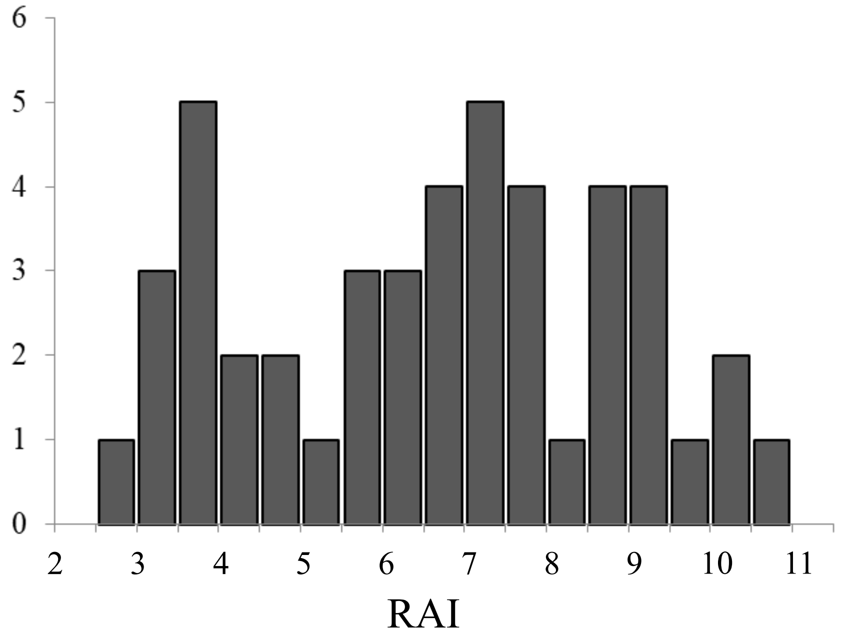

Supplemental Fig. 2 A histogram of RAIs of 46 cultivars.

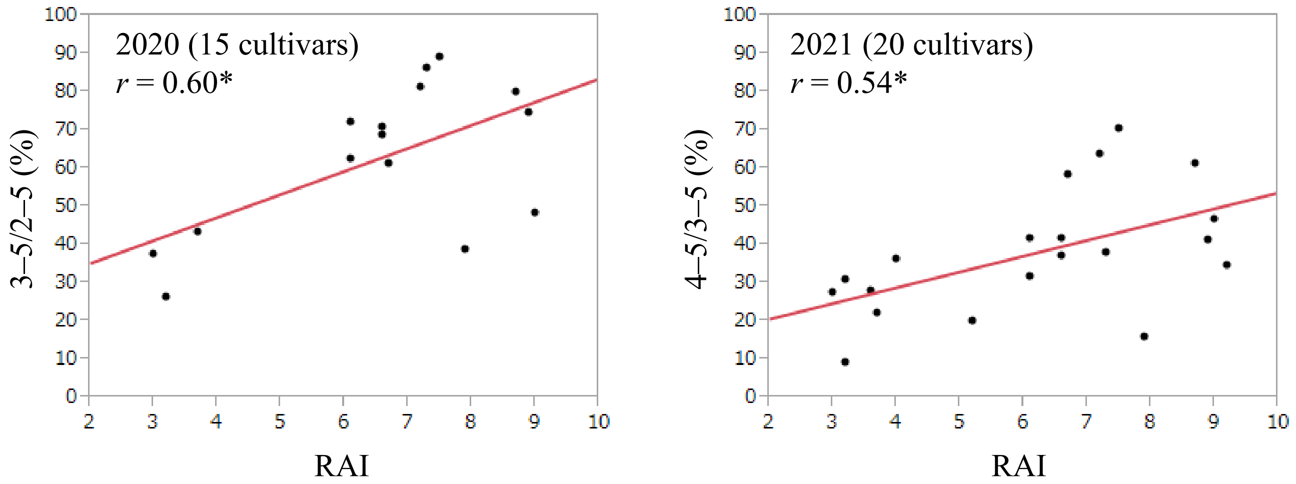

Supplemental Fig. 3 The relationship between RAI in the laboratory test and the proportion of rot index of storage roots in the field test. A red line is a regression line. * $p < 0.05$.
